# An Intelligent Approach for Early and Accurate Predication of Cardiac Disease Using Hybrid Artificial Intelligence Techniques

**DOI:** 10.3390/bioengineering11121290

**Published:** 2024-12-19

**Authors:** Hazrat Bilal, Yibin Tian, Ahmad Ali, Yar Muhammad, Abid Yahya, Basem Abu Izneid, Inam Ullah

**Affiliations:** 1 College of Mechatronics and Control Engineering, Shenzhen University, Shenzhen 518000, China; hbilal@szu.edu.cn; 2 College of Computer Science and Software Engineering, Shenzhen University, Shenzhen 518000, China; ahmadali@szu.edu.cn; 3 School of Computer Science and Engineering, Beihang University, Beijing 100191, China; yarkhan@buaa.edu.cn; 4 Department of Electrical Computer and Telecommunication, Botswana University of Science and Technology Botswana, Plot, Palapye 10071, Botswana; yahyaa@biust.ac.bw; 5 Faculty of Engineering, Department of Robotics and Artificial Intelligence Engineering, Al-Ahliyya Amman University, Amman 19328, Jordan; b.izneid@ammanu.edu.jo; 6 Department of Computer Engineering, Gachon University, Seongnam 13120, Republic of Korea

**Keywords:** cardiac disease, hybrid machine learning techniques, hybrid deep learning approaches, disease prediction, cardiac disease prediction

## Abstract

This study proposes a new hybrid machine learning (ML) model for the early and accurate diagnosis of heart disease. The proposed model is a combination of two powerful ensemble ML models, namely ExtraTreeClassifier (ETC) and XGBoost (XGB), resulting in a hybrid model named ETCXGB. At first, all the features of the utilized heart disease dataset were given as input to the ETC model, which processed it by extracting the predicted probabilities and produced an output. The output of the ETC model was then added to the original feature space by producing an enriched feature matrix, which is then used as input for the XGB model. The new feature matrix is used for training the XGB model, which produces the final result that whether a person has cardiac disease or not, resulting in a high diagnosis accuracy for cardiac disease. In addition to the proposed model, three other hybrid DL models, such as convolutional neural network + recurrent neural network (CNN-RNN), convolutional neural network + long short-term memory (CNN-LSTM), and convolutional neural network + bidirectional long short-term memory (CNN-BLSTM), were also investigated. The proposed ETCXGB model improved the prediction accuracy by 3.91%, while CNN-RNN, CNN-LSTM, and CNN-BLSTM enhanced the prediction accuracy by 1.95%, 2.44%, and 2.45%, respectively, for the diagnosis of cardiac disease. The simulation outcomes illustrate that the proposed ETCXGB hybrid ML outperformed the classical ML and DL models in terms of all performance measures. Therefore, using the proposed hybrid ML model for the diagnosis of cardiac disease will help the medical practitioner make an accurate diagnosis of the disease and will help the healthcare society decrease the mortality rate caused by cardiac disease.

## 1. Introduction

Cardiac disease is among the most common and lethal diseases around the globe, accounting for an average of 17.90% of deaths each year [1]. Cardiac disease has several types, among which cardiovascular disease (CVD) is the most common type, resulting in heart attacks and strokes. In this disease, the heart is generally unable to pump adequate amount of blood to the heart itself and other organs of the body, resulting in a heart attack, which ultimately causes death. In the human body, coronary arteries pump the blood to the heart, and heart attacks occur because of blocking or narrowing of these arteries. Russia has the highest rate of this disease across the globe. According to a report, around 1750 deaths per 100,000 people occur due to cardiac disease. In China, around 931 people per 100,000 people die due to heart disease each year, resulting in a total of 230.0 million morbidities every year. The death ratio due to cardiac disease is expected to increase by 50% from 2023 to 2030 [2]. According to another report published by the European Society of Cardiology, approximately 50% to 55% of cardiac patients die in the initial stages, i.e., 1 to 2.5 years, and the annual cost for the treatment of this disease ranges from 4 to 5% of the annual budget specified for medical care. Heart disease has various symptoms that are common in heart patients, such as an abnormal heartbeat, fatigue, weakness in the body, swollen feet, chest pain, pain in the arms, elbows, or back, dizziness, walking difficulty, severe headache, unconsciousness, etc. The chances of this disease increase by adopting a bad lifestyle, such as a lack of exercise, an unhealthy diet (eating more cholesterol in meals), smoking, drinking alcohol, etc. Doing more exercise, taking part in physical activities, eating a balanced diet, and avoiding smoking and drinking alcohol reduce the chances of heart disease. Adults above 75 years have a higher chance of having cardiac disease as compared to younger people.

These days, CVD is a serious disease in people of all ages in both undeveloped and developed countries [3]. Conventional investigation methods for the diagnosis of CVD are complex and expensive. The earlier approaches for diagnosing CVD were based on the previous medical record, results of the laboratory tests, and doctor examinations. Angiography is the most vital method for identifying heart disease; however, this technique has limitations, like high diagnostic costs and the requirement for an expert cardiologist [4,5]. The earlier heart disease identification techniques sometimes gave inaccurate results due to human mistakes and took more time to assess due to their computationally intensive nature. Furthermore, due to the unavailability of expert medical practitioners and diagnostic resources, especially in developing nations, the diagnosis of heart disease is a very complex process that needs serious attention [6,7,8]. Therefore, the accurate and early prediction of CVD is very important to protect patients from more impairment and assist them in living a healthy and happy life.

Advanced techniques, such as DL and ML, play an important role in the medical sector for diagnosing various diseases. To address the problems present in the earlier invasive-based techniques, researchers started using non-invasive intelligence techniques such as ML and DL to identify cardiac disease. The intelligent ML- and DL-based approaches require no human intervention and consume fewer resources. Moreover, the results produced by the ML and DL approaches are way better than those of the invasive-based techniques. Numerous researchers have used various classical ML algorithms to recognize cardiac disease and achieved satisfactory results. For instance, Van Bussel et al. [9] used linear regression (LR) to identify cardiac disease and achieved a classification accuracy of 77.30%. Similarly, Pandya S., et al. [10] proposed a system for cardiac disease prediction using an infused heart framework and achieved an accuracy of 89.36%. Paul et al. [11] developed a system using adaptive fuzzy rules for cardiac disease, attaining an accuracy of 86.45%. Cohen, K.E., et al. [12] proposed a model based on a hybrid linear method and random forest (RF) algorithm named HRFLM for diagnosing cardiac disease and attained an accuracy of 88.4%.

The earlier non-invasive approaches proposed by various researchers for predicting cardiac disease are mostly based on classical ML and DL algorithms, which perform better than the invasive-based techniques used for diagnosing the mentioned disease. However, there are still some limitations in the classical ML and DL approaches. Firstly, the classical ML and DL approaches have low prediction accuracy, which can lead to the misguidance of the doctor and potentially harm the patient. Secondly, DL-enabled techniques that employ pre-trained models require more resources for model training and validation due to their extensive list of parameters. Thirdly, the lightweight ML and DL models, which are trained from the beginning, cannot interpret the outcomes. Fourthly, current ML and DL models for diagnosing cardiac diseases primarily focus on enhancing accuracy, disregarding the significance of facilitating the interpretation of the model’s output. Therefore, there is an inherent medical risk associated with the utilization of such ML and DL models by healthcare practitioners. The limitations of the classical ML and DL models for cardiac disease diagnosis can be addressed by using novel hybrid ML and DL techniques. Considering the significant performance of the hybrid techniques, this study utilizes different hybrid DL and ML approaches for predicting cardiac disease. The core objective of the proposed hybrid models is to improve the performance of the earlier ML and DL approaches and to develop an intelligent system based on hybrid ML and DL algorithms for predicting cardiac disease, which can accurately diagnose cardiac disease. Our proposed hybrid ML model (ETCXGB) is very lightweight, has fewer parameters, consumes fewer resources, takes much less time for training, and has much higher prediction accuracy compared to the pre-trained models. The contributions of this study are as follows:

- This study proposes a hybrid ETCXGB ML model that is the integration of two ML ensemble learning algorithms, namely ETC and XGB. Further, this study also utilizes three hybrid DL models, namely CNN-RNN, CNN-LSTM, and CNN-BLSTM, for the diagnosis of cardiac disease.
- The proposed ETCXGB hybrid ML model made 99.98% correct predictions by attaining almost 99% values for all the performance evaluation measures. The ETCXGB improved the prediction accuracy by 3.91% for the diagnosis of cardiac disease. Further, the other hybrid DL models also performed well and increased the classification accuracy by 1.95% (CNN-RNN) and 2.44% (CNN-LSTM and CNN-BLSTM).
- The efficiency of the proposed models was measured using both statistical and ML performance measures. Numerous efficiency measures, such as accuracy, sensitivity, specificity, etc., and RMSE, MAE, and MSE, have been used for this purpose. The simulation outcomes depict the significance of the proposed hybrid ML and DL models.

The remaining sections are structured as follows: Section 2 deeply discusses the latest literature on cardiac disease using various ML and DL techniques. Section 3 illustrates the proposed intelligent approach for the early and accurate prediction of cardiac disease. Section 4 shows the experimental setup and environment, while multiple experiments were performed for the diagnosis of cardiac disease using the proposed hybrid ML and DL algorithms. Lastly, Section 5 summarizes the overall theme of this study.

## 2. Literature Review

Cardiac disease is increasing at a great pace all over the world, resulting in an increase in the mortality rate in all continents. Because of its catastrophic nature, this disease has attracted the attention of scholars and medical experts. Various techniques have been used in the past few years for the identification of cardiac disease, which have their own advantages and limitations. Keeping the significant role of ML and DL in mind, different scholars have used them to diagnose multiple diseases such as heart disease, diabetes, stress, pneumonia, brain tumors, etc. [12]. For example, Bashir et al. [13] have used a decision tree (DT) classifier to diagnose cardiac disease by utilizing both information gain and the Gini index. Furthermore, the preprocessing techniques for the removal of outliers and missing values have been used to achieve a diagnosis accuracy of 88.52%. Similarly, Verma et al. [14] proposed a hybrid approach based on DT and MLP for the diagnosis of cardiac disease and achieved an accuracy of 88.40%. With multiple ML classification models, Y. An et al. [15] conducted a comparative analysis for cardiac disease diagnosis and found that some algorithms perform better, but they take longer to train, while others are faster to train, but they do not perform so well. Manogaran et al. [16] have proposed an algorithm-based hybrid system for diagnosing cardiac disease. Multiple experiments were conducted, and promising results were obtained with less data; however, the models’ performance was reduced when more data were provided. ML models have outperformed traditional invasive-based diagnosis methods for predicting cardiac disease, according to Alizadehsani et al. [17]. In addition, it was observed that RF performed better than the other models when comparing their performance.

After the extensive use of classical ML and DL algorithms for the diagnosis of different diseases, the researchers started thinking about the use of hybrid ML and DL models to help the healthcare society [18,19,20]. In this regard, Ali et al. [21] designed a hybrid ML model to identify cardiac disease and achieved 86.20% precision. Numerous preprocessing methods were used for noise removal, the removal of outliers, and data normalization. The Jackknife cross-validation (CV) method was used for the validation of the model’s performance. According to the experimental outcomes, the preprocessing and CV methods helped improve the performance and stability of the model. Similarly, Xie et al. [22] proposed an intelligent model based on the combination of LR and RF for the diagnosis of cardiac disease and achieved an accuracy of 88.69%. The performance of the single models, i.e., LR and RF, were also investigated. However, the performance of the single ML models was not satisfactory. Then, the LR and RF models were integrated to develop a hybrid model, which is an integration of both LR and RF models. After combining both models, it was observed that the hybrid model performed much better than the single ML model. Similarly, Tao et al. [23] proposed an intelligent system using staked SVM for the identification of cardiac disease and achieved an accuracy of 91.10%. To evaluate the proposed staked-SVM model, different kernel types of SVM classifiers, i.e., RBF, linear, sigmoid, and polynomial, were utilized to select the most optimal one. According to the experimental outcomes, the best kernel type that fit the nature of the problem was RBF. The other important parameters of SVM, such as gamma and learning rate, were also tuned properly for better performance of the model.

Dutta et al. [24] have used multiple techniques for the prediction of cardiac disease. Multiple experiments were performed for each investigated ML technique and compared their performance to find out the best technique. After analyzing the performance of the models, it was observed that RF performed better compared to the other models and achieved an accuracy of 89.80%. The selection of an ML model is totally dependent on the specification of the problem. We cannot say that a particular ML model is better than the others; however, it can only be observed once it is used for that problem. Table 1 shows the details of the authors, their proposed methodology, utilized datasets, and the experimental outcomes attained via their proposed method.

Currently, it is the age of cutting-edge hybrid ML and DL approaches that have shown great performance in almost every field of life, particularly in the healthcare sector. Because of the important role of hybrid models in the healthcare sector, this study investigates the techniques mentioned for the diagnosis of cardiac disease. One hybrid ML and three hybrid DL models are proposed to improve the diagnostic systems for cardiac disease. The proposed models include ETCXGB, CNN-RNN, CNN-LSTM, and CNN-BLSTM. These hybrid models perform way better than the classical ML and DL models. Every single model, such as ETC, XGB, CNN, RNN, LSTM, and BLSTM, has its own class and versatility. When these models are combined, their efficiencies are increased further, resulting in higher prediction accuracy as compared to the single model. Among the proposed hybrid models, the ETC-XGB hybrid ML model performed really well and attained the first spot in the performance rivalry. It is anticipated that the ETC-XGB will help the healthcare system make accurate diagnoses of cardiac disease.

## 3. Proposed AI-Based Hybrid Approach for Cardiac Disease Prediction

**Figure 1:**
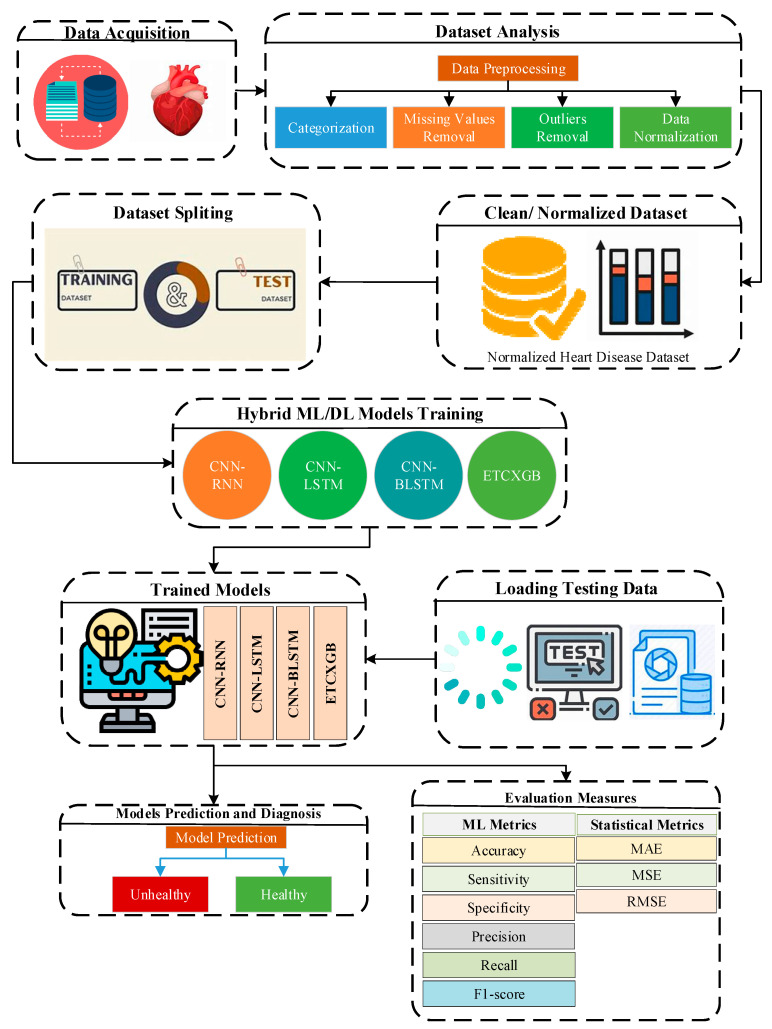
Methodology of the proposed research work.

The proposed AI-based hybrid approach for cardiac disease diagnosis consists of numerous steps. The first step involves selecting a dataset that is more closely associated with the research problem. The second step is to apply various preprocessing techniques to remove missing values and outliers and bring the attribute values into a normalized range and standardized format. The third step presented the normalized data to the proposed hybrid ML and DL models after preprocessing. We used various efficiency measures like precision, recall, and accuracy in the fourth step to check the validity and performance of the suggested algorithms. In the fifth step, the model predicts the status of a patient, whether he or she has cardiac disease or not. Figure 1 represents the proposed work’s complete architecture. The following Sections go into more detail about the data used and the approach used to complete this research study.

### 3.1. Dataset Description

Problem-specific datasets have a great influence on the development and performance of the models. Therefore, the selection of a dataset that has a great association with the target class is of more importance. Multiple datasets are available online for cardiac disease, including the MIT-BIH dataset, the UCI Cleveland heart disease dataset, the Switzerland heart disease dataset, the UCI Hungarian heart disease dataset, etc. In this study, the UCI Hungarian heart disease dataset is used for the diagnosis of cardiac disease. The utilized dataset contains 1025 instances of both males and females. The dataset consists of 13 attributes and a target class. The target class is a binary class that represents healthy (0) and affected (1) or normal (0) and abnormal (1). There are 525 instances that belong to the affected people (1), while the remaining 500 instances contain the data of people who are healthy (0).

### 3.2. Preprocessing Techniques

Using pre-processing methods is a critical step in ensuring the normalization and standardization of datasets, as well as enhancing the efficacy of numerous ML/DL models. Several pre-processing approaches, including categorization, the removal of missing values and outliers, and normalization, prepared the data for the proposed hybrid models. These techniques ensured that the models received the data in a well-organized and standardized format. OneHotEncoder was used to sort out the problem of non-numeric text categorical data into numeric categorical data. Outliers and raw data affect the efficiency of the algorithms; so, missing values were replaced by the mean value, and the outliers were removed properly from the dataset. Another important method is normalizing, or standardizing, the dataset to account for variations in values across distinct columns. In order to offer them to the models in a standardized way, it is necessary to constrain the values within a specific range, specifically from 0 to 1. Consequently, we used an approach known as StandardScaler to address this problem. We noted an improvement in the models’ efficiency after using the specified pre-processing approaches.

### 3.3. Hybrid Extra-Tree Classifier + Xgboost (ETCXGB) ML Model

The ETC is a DT-based ensemble learning method and is an advanced form of the conventional DT algorithm. It builds various DTs in a random style and then integrates their prediction results. The “Extra” in ExtraTree refers to the fact that it generates numerous random splits at every node as opposed to the best split-like conventional DTs. This randomization can enhance generality and make it less prone to overfitting. The ET mainly involves three steps: bootstrap sampling, feature randomness, and split selection. Through a method known as bootstrap sampling, ET makes use of a randomly selected portion of the training data. It generates several data subsets by randomly selecting samples from the training set with replacements. Then, it chooses a random subset from the dataset for each node of the tree. The trees’ diversity is ensured by randomness, which prevents overfitting. Instead of searching through all potential feature values to obtain the optimal split for each node, it chooses various feature thresholds at random and then picks the one that produces the best split based on criteria such as Gini index or entropy. The ET technique is based on the following arithmetic operations:

Random subsampling: This is also known as bootstrap aggregation, in which each cycle begins with the selection of a random subset of the training data to train a DT. We can represent this by selecting a random sample from the training data.

(1) $$S=\{x_{1},\;x_{2},x_{3},\;\ldots ,\;x_{n}\}$$

Equation (1) represents the training set that consists of a total of S samples, where each sample consists of a total of 13 attributes. The arbitrarily chosen subset of the training data is illustrated in Equation (2).

(2) $$S_{i}=\{{xi}_{1},\;{xi}_{2},{xi}_{3},\;\ldots ,\;{xi}_{m}\}$$

Random subsampling reduces overfitting by enabling the model to discover a variety of patterns in the data. The final forecast is more reliable because each tree in the ensemble may pick up somewhat distinct patterns from the identical dataset.

Random subspace: This method introduces additional unpredictability by selecting a random subset of features at each stage of the DT construction process. In this method, each DT randomly chooses a subset of attributes. The arbitrarily chosen subset of the feature space can be used as an illustration of it and is given as follows:

(3) $$F=\{f_{1},\;f_{2},f_{3},\;\ldots ,\;f_{n}\}$$

In the above Equation, F illustrates the total features of the training samples, whereas $f_{1}$ represents the first feature, and $f_{n}$ notifies the 13th feature of the training samples.

Complete feature-space is depicted in Equation (3), whereas the casually selected subset of attributes is depicted in Equation (4).

(4) $$F_{i}=\{{fi}_{1},\;{fi}_{2},{fi}_{3},\;\ldots ,\;{fi}_{k}\}$$

The fact that different *DT*s use different subsets of attributes makes the *DT*s more diverse. The *DT*s with lower correlations are more diversified, which enhances generalization and lowers the chance of overfitting. For heart disease prediction, the model will perform better on unseen data because it would not be overly sensitive to a single feature of the heart disease dataset.

Tree generation: The *DT* method employs a specific subset of training data and carefully selected characteristics to create a *DT*. We can express this as a function that receives a portion of training features and data as input and generates a *DT* as an output.

(5) $$T_{i}=DT\left(S_{i},\;F_{i}\right)$$

*DT*s, which are not well-matched to certain features or criteria, are the result of this unpredictability. This increases the model’s resilience for heart disease prediction by reducing its reliance on a particular feature threshold.

Aggregation: In order to create a prediction, the ensemble tree (ET) combines the predictions made by each individual tree. A method that receives the set of *DT*s as an input and generates a final prediction can express this.

(6) $$Y=Aggregation(T_{1},\;T_{2},\;,\;T_{3},\;\ldots ,\;T_{k})$$

Several approaches, such as voting by majority or weighted polling, can accomplish the aggregate function.

An improved gradient-boosting (GB) technique noted for its performance and scalability is called XGBoost (XGB). By merging the predictions of numerous weak models, typically DTs, GB is an ensemble learning technique that creates a powerful predictive model. Regularization, the management of missing values, and the use of a more effective optimization technique are all ways that XGB improves on conventional GB. The mathematical representation of this algorithm is given as follows:

XGBoost constructs a model F(x) that optimizes a specific objective function given a training set of n examples {(*x*_1_, *y*_1_), (*x*_2_, *y*_2_),…, (*x_n_*, *y_n_*)}, where xi represents the input characteristics and *y_i_* represents the output label:

(7) $$L\left(F\right)=\sum_{k=0}^{n}l\left(F\left(x_{i}\right),\;y_{i}\right)+\;\Omega \left(F\right)$$

Equation (7) defines the loss function, denoted by *l*, which calculates the inconsistency between the predictable and genuine result. The regularization term, Ω, restricts models that are overly complex. *F*(*x_i_*) represents the predicted result for the input xi. XGBoost employs gradient boosting to incrementally incorporate decision trees into the model. Each tree is trained using the residuals of the previous tree, and the ultimate model is the sum of all the trees. Equation (8) represents the model’s detection rate for a new input *x*.

(8) $$F\left(x\right)=\sum_{k=0}^{n}T_{k}(x)$$

where $T_{k}\left(x\right)$ is the prediction of the kth tree.

**Figure 2:**
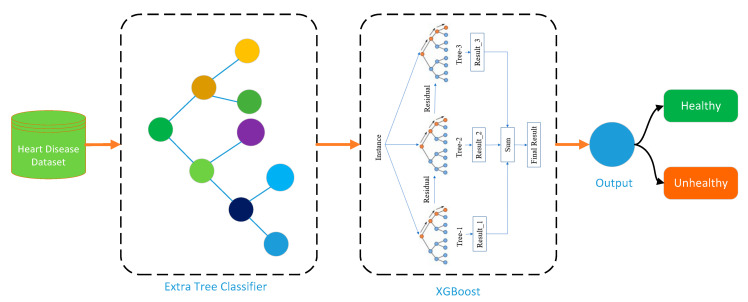
Structure diagram of ETCXGB hybrid ML model.

A single model may not always perform as well as a combination of numerous models. The purpose of merging ETC and XGB is to take advantage of each algorithm’s capabilities. The combination of these models can result in a versatile and accurate hybrid ML model that can produce more accurate results than the single ETC and XGB algorithms. Figure 2 represents the proposed ETCXGB hybrid ML model.

The proposed model integrates the output of the ETC ensemble model with the XGB model, where the output of ETC is used as an input for the XGB model. It can be represented mathematically as follows:

(9) ETC−XGB (x)=Prediction (XGB)

In the proposed model, the ETC model provides a prediction in binary class, i.e., whether a patient has the cardiac disease or not. The prediction results are then passed to the XGB model, which further refines it by adding boosting trees to the ETC results. The proposed ETCXGB model was implemented in the Anaconda Jupyter Notebook IDE using TensorFlow at the backend, while the programming language used was Python. The simulations were carried out. The system specifications, such as the system model, RAM, processor, etc., are discussed in more detail in Section 4. As parameter setting plays a key role in ML and DL, we tuned the parameters on a regular basis and finally chose the optimal parameter values for each parameter. The main parameters and values of the ETC model include n_estimators: 100, criterion: gini, max_features: auto, min_samples_split: default, min_samples_leaf: default, and bootstrap: false. The optimal parameter values chosen for the important parameters of the XGB model include n_estimators: 100, learning_rate: 0.05, max_depth: default, min_child_weight: 1, subsample: default, gamma: 0.0001, lambda: default, alpha: default, and booster: gbtree. The training and testing setting of the dataset was opted as 70:30, which means 70% of the data was used for training and 30% was used for testing the model. To avoid overfitting, we also make use of the k-fold cross-validation technique, using the value of k as 10 (10-fold). The ETC-XGB model’s final prediction is a binary value that depicts the likelihood of the positive (1) and negative (0) classes, where the positive class represents the presence of cardiac disease, while the negative class illustrates the absence of cardiac disease. This hybrid technique uses the flexibility of the initial ensemble (ETC) and the continuous correction of errors during boosting (XGB) to improve the accuracy of predictions, especially for binary classification problems. Also, the ETC-XGB model consumes fewer resources, takes less time in training and testing, and provides higher prediction accuracy compared to the earlier models used in the literature.

### 3.4. Proposed CNN-RNN Hybrid DL Model

CNN is one of the most popular and commonly used DL algorithms. CNN has numerous applications in different fields, such as robotics, agriculture, transportation, education, medical care, etc. CNN has been extensively utilized in the medical field due to its versatile and reliable nature. CNN employs feature extraction techniques to derive valuable characteristics from the data, enabling a comprehensive understanding of its underlying nature [36,37]. It comprises convolutional layers, pooling layers, and activation functions. The convolutional layer uses kernels (filters) for the input data that detect particular patterns in the input data.

After identifying specific features in the input data, it outputs a set of feature maps that illustrate different classes of data. Let us suppose R is the number of rows, C is the number of columns in a data set, G is the number of channels, K is the filter size, and F is the number of filters used in a layer. Then, the resultant feature map for every filter is computed via the following formula:

(10) $$Y_{i,j}=\sum_{x=1}^{K}\sum_{y=1}^{K}\sum_{g=1}^{G}W_{x,y,g\;}^{F}X_{i+x-1,j+y-1,\;g\;}$$

where $Y_{i,j}^{F}$ illustrates the feature map value for the *f*-th filter at position (*i*, *j*). $W_{x,\;y,g}^{F}$ shows the weight of the *f*-th filter at position (*x*, *y*) and channel *g*. $X_{i+x-1,j+y-1,g}$ depicts the input value at position (*i* + *x* − 1, *j* + *y* − 1) and channel *g*. In order to introduce non-linearity into the model, an activation function is added to each element, following the convolution procedure. ReLue is one of the popular activation functions that is used most and is given as follows:

(11) $$Relue\;\left(a\right)=Max\left(0,\;a\right)$$

The pooling layer preserves important information while shrinking the feature maps’ spatial dimensions. Max pooling is a popular technique in which each feature map’s output is the maximum score found in a small area:

(12) $$Y_{i,j}^{F}={Max\;(X}_{2i,2j\;}^{F},\;X_{2i,\;2j+1}^{F},\;X_{2i+1,2j}^{F},X_{2i+1,\;2j+1}^{F}$$

In Equation (12), $Y_{i,j}^{F}$ represent the pooled feature map value for the *f*-th filter for *Y*(*i*,*j*).

The RNN is one of the important DL algorithms that is particularly used to handle sequential data. In RNN, recurrent connections are used to simulate interdependence across time. The hidden state is the central part of an RNN that becomes revised based on the input at every interval as well as the prior hidden state. The hidden state of RNN can be represented as follows:

(13) $$H\left(t\right)=RNN_{c}\left(x_{t},\;h_{t-1}\right)\;$$

In Equation (13), $h_{t}$ signifies the hidden state at time *t*, $RNN_{c}$ shows the RNN cell, $x_{t}$ illustrates the current input, while $h_{t-1}$ depicts the previous hidden state. Different RNN cells are used, among which vanilla is the most commonly used one. The vanilla $RNN_{c}$ can be computed as follows:

(14) $$H\left(t\right)=Tan\left(W_{hth}h_{t-1}+W_{xh}x_{t\;}+b_{h}\right)$$

**Figure 3:**
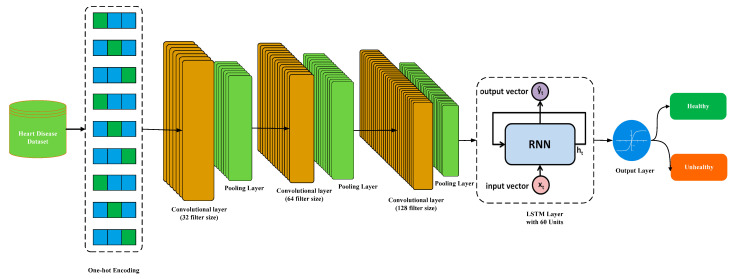
A structure diagram of the CNN-RNN DL model.

Figure 3 represents the architecture of the proposed CNN-RNN hybrid DL model. The CNN-RNN model combines the sequential information modeling of RNNs with the spatial feature extraction skills of CNNs to deal with problems that need both spatial and time-series data. A series of feature maps represent the CNN output in CNN-RNN. The output of CNN is then used as an input for the RNN model, which processes it in order to comprehend the temporal dynamics. The input of 13 features of the heart disease dataset is given as the input to the CNN model. The CNN model extracts spatial features from the input data and performs the necessary data processing to bring them into a format that best suits the RNN model. The CNN part consists of three convolutional layers: the first convolutional layer uses a filter size of 32, the second convolutional layer utilizes a filter size of 64, and the third layer uses a filter size of 128. All three layers use a kernel size of 3 and an activation function called “relu”. A pooling layer (MaxPooling) of size 2 follows all the convolutional layers. Similarly, the RNN part uses a filter size of 64, an activation function of “tanh,” and a return sequence of 1. A dropout layer follows, maintaining a dropout value of 0.2. The second layer of the RNN consists of 32 filters, and the activation function used is “tanh”. The output layer comprises a dense layer with a constant value of 1 and employs the “sigmoid” activation function. The optimizer uses “adam” for model compilation and “binary_crossentropy” for the loss function. We keep the batch size at 32 and the number of epochs at 150 for training the model.

The main objective of the proposed hybrid model is to fuse the features of CNN and RNN and use them for the early diagnosis of cardiac patients. There are various fusing methods for combining the features of both DL models; however, we used the fully connected layer technique to fuse the features of both CNN and RNN. The fusion of CNN and RNN can be represented mathematically as follows:

(15) $$CNN-RNN\;(HF)=Concate(Output\left(CNN\right),H_{t})$$

In Equation (15), HF represents the hybrid features, which illustrates the integrated feature vector. The concate function is used to concatenate the features extracted by the CNN and the RNN ($H_{t})$. The hybrid-feature vector consists of the information from the cardiac patient data, extracted via CNN and processed by the RNN.

The last step of the CNN-RNN model (hybrid features vector) is to determine whether the patient is healthy or has cardiac disease. This process is accomplished by the fully connected layer, followed by the activation function (Softmax), which is more suitable for binary class prediction.

(16) $$Prediction=Softmax(FC\left(HF\right))$$

In Equation (16), *HF* represents the hybrid features, and *FC* illustrates the fully connected layer. The Softmax activation function is used to transform the output of the model into the likelihood of a cardiac or healthy patient. In order to learn the best parameters for precise diagnosis, the model is trained using labeled cardiac data. The performance of CNN-RNN shows its significance for the diagnosis of cardiac disease.

### 3.5. Proposed CNN-LSTM Hybrid DL Model

A DL architecture that combines CNN and LSTM is known as the CNN-LSTM model. Tasks involving both sequencing data, such as time series or sequencing text data, and spatial data, such as images, are ideally suited for this hybrid paradigm. In CNN-LSTM, the CNN part extracts useful spatial features from the data, while the LSTM part is used to handle sequential information modeling. The working of CNN, its applications in different fields of life, and its mathematical representation were already explained in more detail in Section 3.4.

**Figure 4:**
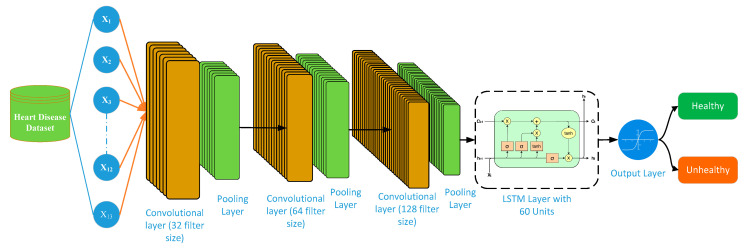
Structure diagram of CNN-LSTM DL model.

A kind of RNN called an LSTM network is made to describe sequential data and manage long-term dependencies. It fixes the vanishing gradient issue that plagues conventional RNNs, which enables them to capture data across longer sequences. The main component of an LSTM network is the LSTM cell, which manages and maintains three important gates: the input, forget, and output gates. The flow and control of information within the cell are the responsibility of these gates. The LSTM cell’s hidden state, which also extracts significant information from the data sequence, serves as a representation of the memory. Figure 4 represents the proposed CNN-LSTM hybrid DL model architecture.

For a single time-step (t), the LSTM cell equations can be described as follows:

For input gate:

(17) $${In}_{t}=\delta (W_{xin}\;*\;x_{t}+W_{hin}\;*\;h_{t-1}+b_{in})$$

The amount of new data that is saved in the cell state is determined by the input gate. In Equation (17), ${In}_{t}$ illustrates the input at time t, δ notifies the activation function, $x_{t}$ is the input at timestep t, $h_{t-1}$ represents the previous hidden state at timestep t−1, whereas $W_{hin}$ depicts the weights, and $b_{in}$ describes the bias at the input gate.

For forget gate:

(18) $${Fo}_{t}=\delta (W_{xfo\;}*x_{t}+W_{hfo}*h_{t-1}+b_{fo})$$

The amount of the prior cell state that should be forgotten is determined by this gate. In Equation (18), ${Fo}_{t}$ illustrates the forget state at time t, δ notifies the activation function, $x_{t}$ is the input at timestep t, $h_{t-1}$ represents the previous hidden state at timestep t−1, whereas $W_{hfo}$ depicts the weights at the hidden state, and $b_{fo}$ describes the bias at the forget gate.

For output gate:

(19) $${Ou}_{t}=\delta (W_{xou\;}*x_{t}+W_{hou}*h_{t-1}+b_{ou})$$

This gate identifies the output of the hidden state at timestep t, which is used for the prediction of the cardiac disease. In Equation (19), ${Ou}_{t}$ illustrates the output of the output gate at timestep t, δ notifies the activation function, $x_{t}$ is the input at timestep t, $h_{t-1}$ represents the previous hidden state at timestep t−1, whereas $W_{hou}$ depicts the weights at the hidden state, and $b_{ou}$ describes the bias at the output gate.

For the update of cell state, we have the following:

(20) $$C_{t}=Tanh(W_{xc\;}*x_{t}+W_{hc}*h_{t-1}+b_{c})$$

In updating the cell state, the cell state is updated by integrating the previous and new cell state. In Equation (20), $C_{t}$ illustrates the cell state, while the Tanh represents the activation function. The rest of the parameters are the same as other states.

For the hidden state, we have the following:

(21) $$h_{t}=O_{t}.Tanh(C_{t})$$

In the above Equations, $x_{t}\;$ represents the input, and $h_{t}\;$ depicts the hidden state at time t, δ illustrates the activation function (sigmoid), W shows the weight, while b notifies the bias. The hidden state illustrates the output of the LSTM cell that is utilized for the prediction of the cardiac disease.

An input of 13 features of heart disease is given as input to the CNN part of the CNN-LSTM hybrid DL model as shown in Figure 4. The CNN part of CNN-LSTM extracts spatial features from the input data. The proposed CNN architecture consists of three convolutional and three pooling layers. The convolutional layers extract useful features from the data, while the pooling layers fuse the learned features of CNN. The first convolutional layer uses a filter size of 32, a kernel size of 3, and an activation function as a relu. A pooling layer precedes the convolutional layer. We have used MaxPooling in our study by keeping the pool_size at 2. The second convolutional layer uses a filter size of 64, a kernel size of 3, and an activation function called relu. Like the first and second convolutional layers, we have used the third convolutional layer by keeping its filter size at 128, its kernel size at 3, and its activation function at relu. A pooling layer of size 2 follows the third convolution layer, just as it does for the other layers. All layers maintain a constant pooling layer size of 2 × 2. By reducing the number of parameters and network calculations, the pooling layer shrinks the spatial dimension. Spatial features, the CNN’s output, serve as the input for the LSTM. The LSTM processes the input and generates an output that aids in the identification of cardiac patients. The LSTM layer has a filter size of 64 and uses the activation function “Tanh”. The output layer uses a dense layer with a filter size of 1 and an activation function called “sigmoid”. The model is compiled using the optimizer “adam” and the loss “binary_crossentropy”. For the model training, it uses a batch size of 64 and a number of epochs of 150.

### 3.6. Proposed CNN-BLSTM Hybrid DL Model

A DL architecture that combines CNN and BLSTM networks is known as the CNN-BLSTM model. CNN-BLSTM is made to handle jobs requiring sequential and spatial data, like time series or sequential text data, as well as spatial data, such as images. The CNN part extracts spatial features from the data, while the BLSTM part captures sequential relationships in both forward and backward directions. The working of CNN, its applications, and its mathematical representation were already discussed in Section 3.4.

A particular class of RNN called BLSTM networks is capable of capturing sequential interdependence in both the forward and reverse directions. The BLSTM effectively captures long-term dependencies in sequential data by fusing the benefits of forward and backward LSTMs. The BLSTM cell is the central element of BLSTM. Two independent LSTM networks, one interpreting the sequence in the forward and the other processing in the reverse direction, make up the BLSTM cell. The final result is the combination of the two LSTM networks. There are two types of hidden states in BLSTM, known as forward and backward hidden states. The hidden states of BLSTM are a combination of both of these states.

(22) $$H_{BLSTM}=\left[H_{for},\;H_{back}\right]\;$$

The forward LSTM cell’s formulas are exactly the same as those that were mentioned in the CNN-LSTM description. The formulas for the backward LSTM cell are also the same as those for the forward, but they have different weights and biases.

For the input gate:

(23) $$I_{t}^{back}=\delta (W_{xi}^{back}*x_{t}+W_{hi}^{back}*h_{hi}^{back}+b_{i}^{back})$$

For the forget gate:

(24) $$F_{t}^{back}=\delta (W_{xf}^{back}*x_{t}+W_{hf}^{back}*h_{hf}^{back}+b_{f}^{back})$$

For the output gate:

(25) $$O_{t}^{back}=\delta (W_{x0}^{back}*x_{t}+W_{ho}^{back}*h_{ho}^{back}+b_{o}^{back})$$

For the update of the cell state, we have the following:

(26) $${\;C}_{t}^{back}=Tanh(W_{xc}^{back}*x_{t}+h_{hc}^{back}*h_{t+11}^{back}+b_{c}^{back})$$

For the hidden state, we have the following:

(27) $$h_{t}^{back}=O_{t}^{back}\;\;Tanh\;(C_{t}^{back})$$

The working process of both forward and backward steps is the same, but the way of processing the input sequence is different. The forward layer processes the sequence of data in a forward direction, i.e., from left to right, whereas the backward layer processes it in a backward direction, i.e., from right to left. The final output of the BLSTM is the concatenation of both forward and backward hidden states. At each timestep t, the output of BLSTM is given as follows:

(28) $$h_{t}^{BLSTM}=h_{h}^{For}\times h_{h}^{Back}$$

**Figure 5:**
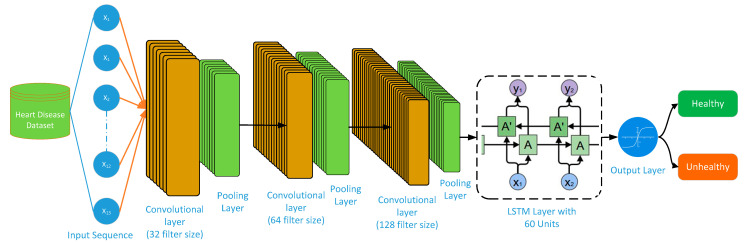
Structure diagram of CNN-BLSTM DL model.

The CNN-BLSTM model integrates the ability of CNNs to extract spatial features with the time-dependent modeling abilities of BLSTMs. Figure 5 depicts the architecture of the proposed CNN-BLSTM model.

The following steps are involved in the proposed CNN-BLSTM architecture:

- The CNN part of the CNN-BLSTM is used to initially process the input cardiac data and extract spatial information from it. The input data contain a total of 13 features of UCI Hungarian heart disease dataset.
- The CNN part of CNN-BLSTM processes the input data and produces a series of feature maps from the input data.
- A series of feature maps from the CNN are sent to the BLSTM part as its output, where they are used as input for the BLSTM.
- The sequential feature maps are processed in both directions by the BLSTM, preserving both backward and forward temporal correlations.
- The BLSTM’s output is the identification of a patient status, that is, whether the patient has cardiac disease or not.

Like the CNN-LSTM model shown in Section 3.6, an input of 13 features of heart disease is given to the CNN part of the CNN-BLSTM hybrid DL model. The number of convolutional layers and their filter size, as well as the number of pooling layers and their kernel sizes, are the same for both CNN-LSTN and CNN-BLSTM.

### 3.7. ML and Statistical Performance Metrics

Performance metrics are used for measuring the performance of ML and DL models. A research scholar cannot say in advance that one ML or DL model is better than the other, until its outcomes are measured in terms of different performance metrics. These metrics are very crucial for the selection of a model for solving a particular problem. Here is a brief description of the considered ML and statistical metrics:

Accuracy: Accuracy refers to the proportion of correct identification made by an ML model. It is calculated by dividing the number of properly forecasted observations by the total number of forecasted events across all classes. The accuracy value falls within the range of 0 to 100. A value of 0 signifies an inaccurate prediction by the algorithm, while a value of 100 signifies a correct prediction. This computation can be performed using Equation (29).

(29) $$\mathrm{Accuracy}=\frac{TP+TN\;}{TP+TN+FP+FN}\times 100\%$$

Sensitivity: Sensitivity refers to the ability of a model to accurately identify positive examples in a dataset. It is also known as the true positive rate (TPR). Put simply, sensitivity is the ability of a model to accurately identify positive samples. It is important to know that the sum of the TPR and the false negative rate (FNR) will always be equal to 1. A higher TPR indicates a more precise detection of the positive samples. It can be computed as follows:

(30) $$\mathrm{Sensitivity}=\frac{TP}{TP+FN}\times 100\%$$

Specificity: Specificity is the ability of a model to accurately identify the true negative cases in a dataset. In this scenario, the negative instances correspond to healthy people. Therefore, when the model correctly predicts a healthy person as a healthy individual, it demonstrates the model’s specificity. Here’s the formula for calculating specificity:

(31) $$\mathrm{Specificity}=\frac{TN\;}{TN+FP}\times 100\%$$

Precision: Precision is determined by dividing the total number of properly classified positive occurrences (*TP*) by the percentage of all detected positive tests (whether correctly or erroneously). Precision can be computed as follows:

(32) $$\mathrm{Precision}=\frac{TP}{TP+FP}*100\%$$

F1-Score: The F1-score, a significant metric for evaluating performance in both binary and multiple-class situations, is the mathematical average of recall and precision. When the dataset exhibits skewness, the F1-score becomes more significant than accuracy. Therefore, the F1 metric is crucial in assessing the model’s performance in this scenario. The F1 score typically ranges from 0 to 1. We can compute the F1-score using Equation (33).

(33) $$\mathrm{F1}‐\mathrm{Measure}=\frac{2*(Precision*Recal)}{Precision+Recall}*100\%$$

MCC score: The Matthews correlation coefficient (MCC) is a crucial metric for assessing the efficacy of a model. Its function is to assess or compute the disparity between the anticipated and actual values. The MCC is a numerical measure that ranges from −1 to +1. A value of −1 represents poor performance of the algorithm, while a value of +1 reflects the optimum effectiveness of the model. The model’s MCC can be computed using Equation (34).

(34) $$\mathrm{MCC}=\frac{TP\times TN-FP\times FN}{\surd (TP+FP)(TP+FN)(TN+FP)(TN+FN)}\times 100\%$$

Mean square error (MSE): The MSE is a statistical metric that quantifies the mean or average value of the squared differences between the observed and projected outcomes of a given model. The value of MSE depends on the error rate of the model; the MSE of a model is 0 when the model makes a 100% correct prediction. Its value increases when the model makes incorrect predictions. MSE is also called mean-squared deviation. The MSE of a model can be computed as follows:

(35) $$MSE=\frac{1}{n}\sum_{i=1}^{n}{\;\left(y_{i}-y_{i}\right)}^{2}$$

Mean absolute error (MAE): Absolute error (AE) refers to the amount of error that occurs during the prediction process. Actually, it is the discrepancy between the projected and actual values. Whereas MAE is the mean of the absolute errors and is calculated as follows:

(36) $$MAE=\frac{1}{n}\sum_{i=1}^{n}\left|x_{i}-x\right|\;$$

Root mean square error (RMSE): The RMSE calculates the mean difference between the actual and projected values of a model. RMSE is the standard deviation of residuals. Residuals illustrate the displacement between the data points and the regression line. RMSE calculates the residuals’ degree of dispersion and shows how closely the observed data clusters around the predicted values. RMSE is mathematically represented as follows:

(37) $$RMSE=\sqrt{\frac{1}{n}\sum_{i=1}^{n}{\;\left(y_{i}-y_{i}\right)}^{2}\;}$$

## 4. Simulation Results and Discussion

The non-invasive hybrid ML and DL techniques proposed for the diagnosis of cardiac disease are discussed in more detail in this Section. First of all, the environment for code generation and experimentation was selected. Anaconda Navigator was selected as an integrated development environment (IDE) and Python as a language for the training and testing of the proposed hybrid ML and DL models. All the experiments were conducted on a computer system with the following specifications: 8 GB RAM, 256 GB SSD, 500 GB HD, HP ProBook 840 G4, Core i7 8th generation. Furthermore, multiple experiments were performed for all of the proposed models, and their performances were tested using numerous ML and statistical performance metrics. The following subsections represent a thorough overview of the experimental outcomes of the proposed hybrid ML and DL models.

### 4.1. Performance of Proposed ETCXGB Hybrid ML Model

**Figure 6:**
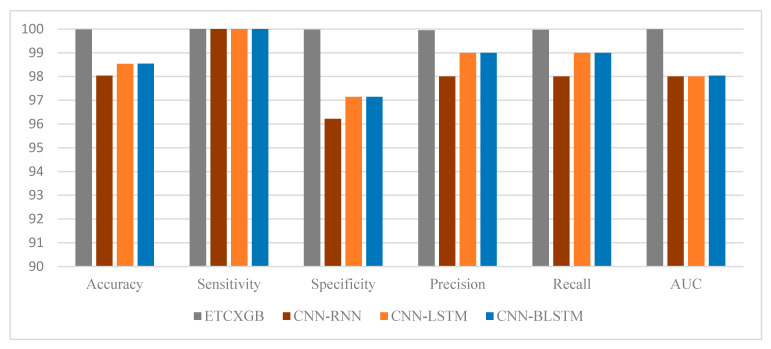
Results of the investigated hybrid models in terms of ML performance measures.

**Figure 7:**
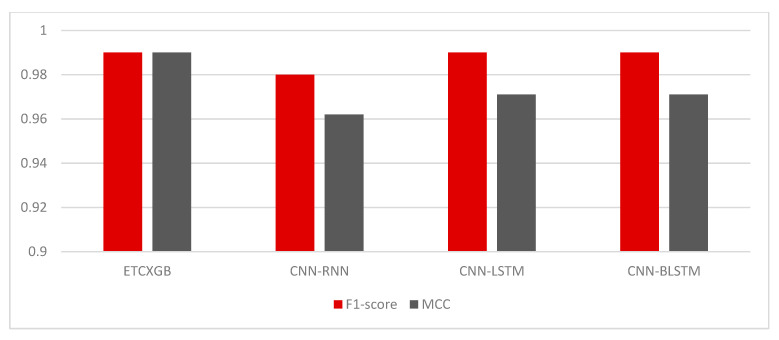
Results of the utilized hybrid models in terms of F1 and MCC scores.

ExtraTree Classifier (ETC) is among the most popular and vital ML classification algorithms. It performs very well on binary class data. Similarly, XGBoost (XGB) is another popular ML classification algorithm that performs really well on both binary and ternary class data. Combining both of these algorithms results in a hybrid ML model that performs much better than the single ETC or XGB algorithm. This Section shows the experimental results of the ETCXGB hybrid ML models using the heart disease dataset. The proposed hybrid ETCXGB performed effectively in terms of all ML performance measures, such as accuracy, precision, recall, sensitivity, specificity, AUC, F1-score, and MCC, as shown in Table 2 and Figure 6 and Figure 7. The proposed ETCXGB has performed efficiently by achieving 99.98% of accuracy, 100% sensitivity, 99.97% specificity, 99.95% precision, 99.96% recall, and 99.99% AUC, as well as an F1-score and MCC of 0.99 for the ML performance measures that were looked into. This means that the proposed ETCXGB model made a 100% correct prediction for the cardiac disease diagnosis.

**Figure 8:**
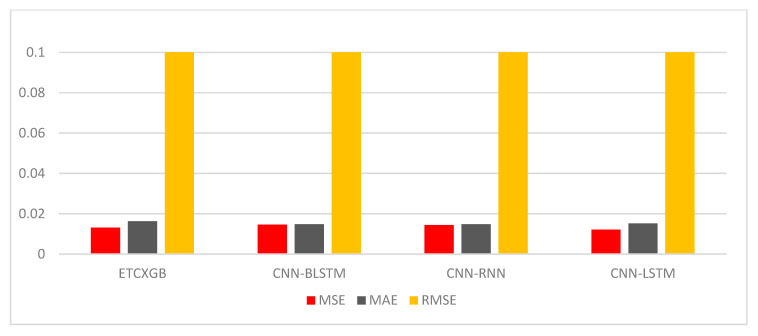
Results of the utilized hybrid models in terms of statistical measures.

**Figure 9:**
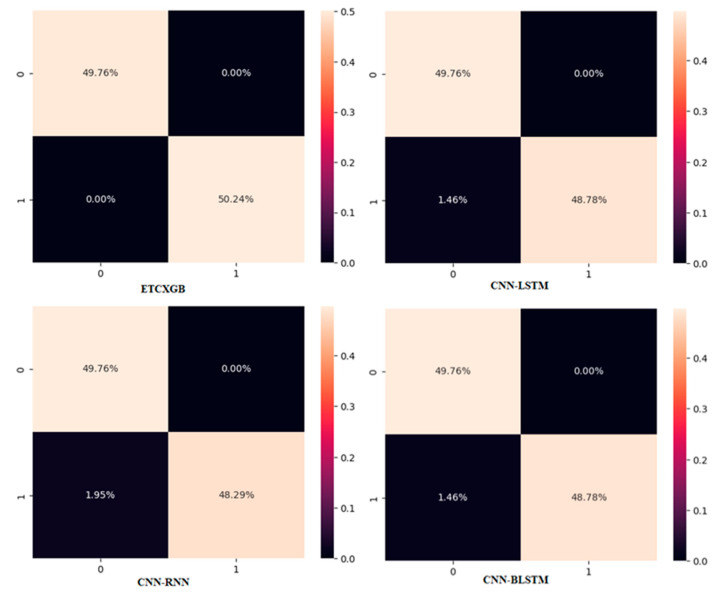
Confusion matrix of the utilized hybrid models.

**Figure 10:**
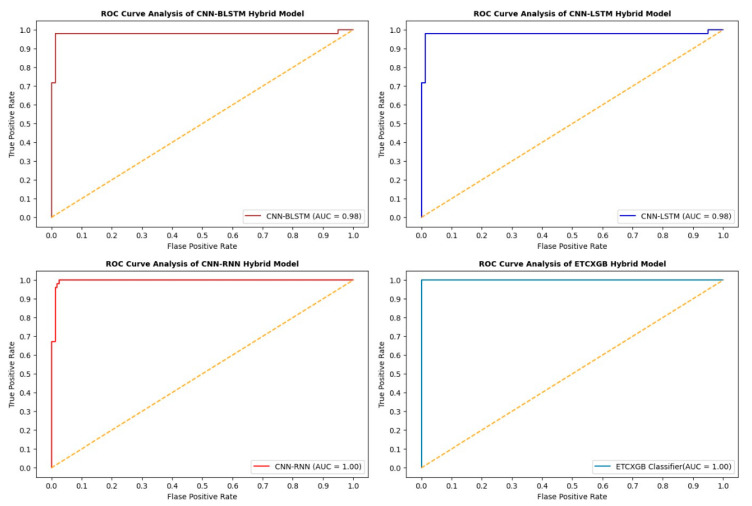
Results of the utilized hybrid models in terms of ROC curves.

Besides numerous ML performance measures, various statistical performance measures such as MSE, MAE, and RMSE were also utilized to check the performance of the proposed ETCXGB hybrid ML model. According to the experimental results, the proposed model also showed good performance in terms of all statistical measures. The MSE value was computed as 0.0121, the MAE value was calculated as 0.0152, and the RMSE value was observed as 0.1102 for the proposed ETCXGB hybrid ML model, as shown in Table 2 and Figure 8. The ML and statistical performance measures depicted in Table 2 show the efficiency and vitality of the proposed models. The ROC curve and confusion matrix are also two important performance measures that play a vital role in tracking the performance of a model and help in selecting the most prominent model from a set of models for a particular problem. Along with the above ML and statistical performance measures, the confusion matrix and ROC curve for the proposed ETCXGB hybrid ML model were also computed and are shown in Figure 9 and Figure 10, respectively.

### 4.2. Performance of Proposed CNN-RNN Hybrid DL Model

CNN’s effectiveness and importance cannot be disregarded, as it has brought a revolution in the medical field due to its reliable performance. Similarly, for data involving sequencing and short-term storage, the performance and role of RNNs are of great significance. When these techniques are applied separately to a problem, it performs admirably; however, its performance improves when these techniques are integrated into a single model to form a hybrid model.

This Section represents the results attained via the proposed CNN-RNN hybrid DL model. We tuned all the important parameters of the CNN and RNN models, i.e., the proposed CNN-RNN model, and performed multiple experiments. After performing multiple experiments, the fine-tuned parameters were selected, and the best performance of the proposed CNN-RNN hybrid DL model was observed as recorded in Table 2. The performance of the CNN-RNN hybrid DL model was also tracked in terms of different statistical performance measures such as MSE, MAE, and RMSE, as shown in Table 2.

The CNN-RNN hybrid deep learning model exhibited exceptional performance across all machine learning evaluation metrics. It had an accuracy of 98.04%, sensitivity of 100%, specificity of 96.22%, precision of 98.00%, recall of 98.00%, AUC score of 98.00%, f1-score of 0.98, and MCC of 0.962, as shown in Table 2 and Figure 6 and Figure 7. The CNN-RNN hybrid DL model also showed great efficiency in terms of the investigated statistical performance measures, such as MSE, MAE, and RMSE. The CNN-RNN accounted for the MSE value of 0.0144, MAE value of 0.0148, and RMSE value of 0.1200, as shown in Table 2 and Figure 8. The confusion matrix and ROC curve of the CNN-RNN hybrid DL model are shown in Figure 9 and Figure 10.

**Figure 11:**
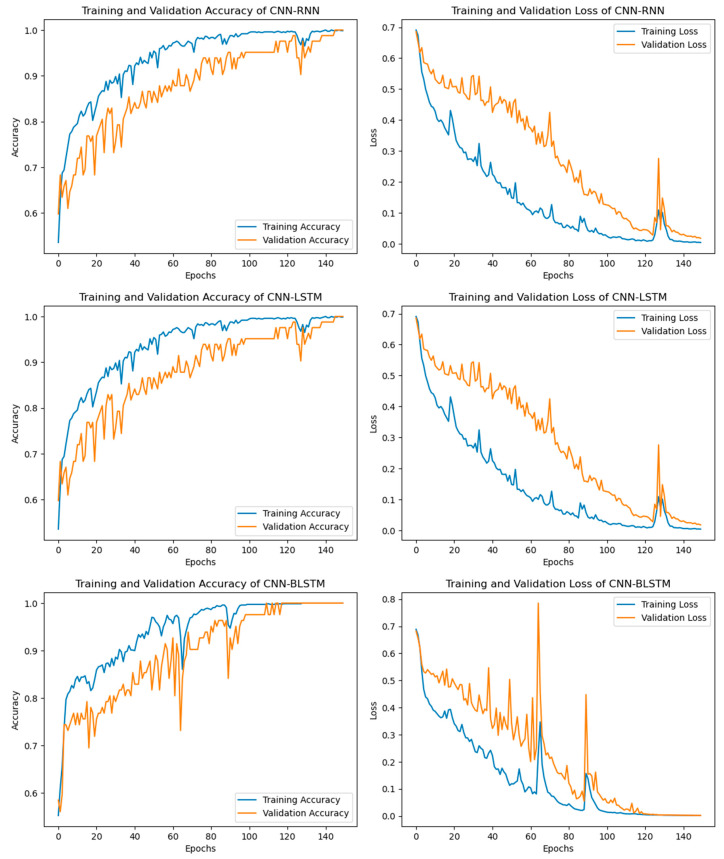
Training and validation accuracies and losses of the proposed hybrid models.

In addition to the ML and statistical performance measures in Table 2, the proposed CNN-RNN hybrid DL model’s training and validation accuracy and loss were also calculated, as shown in 11. Figure 11 shows that as the number of epochs goes up, the CNN-RNN hybrid DL model gets better at both training and testing accuracy. Also, the training and validation losses of the CNN-RNN decrease with the increase in the number of epochs. The highest training and validation accuracies were observed for epoch number 150, while the lowest training and validation losses were calculated at epoch number 150, as shown in Figure 11.

### 4.3. Performance of Proposed CNN-LSTM Hybrid DL Model

CNN and LSTM are widely employed and highly adaptable deep learning techniques. These algorithms have their own significance in different fields of life, particularly healthcare. To benefit from these algorithms, this study utilized the combination of these models to improve the identification rate of cardiac disease. At first, CNN and LSTM were utilized separately, and the experimental outcomes of both CNN and LSTM models were noted. Then, the combination of these two models (hybrid model) was used for cardiac disease diagnosis and measured its outcomes. The experimental outcomes of the CNN-LSTM were measured using both statistical and ML performance measures. After evaluation, it was observed that the hybrid model performed much better than the single models, as shown in Table 2 and Figure 6, Figure 7 and Figure 8.

Table 2 and Figure 6 and Figure 7 illustrate that the hybrid model performed brilliantly in terms of all ML performance evaluation metrics. The CNN-LSTM attained an accuracy of 98.53%, a sensitivity of 100%, a specificity of 97.14%, a precision of 99.00%, a recall of 99.00%, an AUC score of 98.00%, an F1-score of 0.99, and a MCC score of 0.971. Likewise, the MSE value for the CNN-LSTM was computed as 0.0121, the MAE value was calculated as 0.0152, and the RMSE value was observed as 0.1102, as shown in Table 2 and Figure 8.

The confusion matrix and ROC curve of the proposed CNN-LSTM model are shown in Figure 9 and Figure 10. Calculating the effectiveness of the studied model also relies heavily on the training and validation losses and precisions achieved by the CNN-LSTM hybrid DL model. Figure 11 shows the training and validation accuracies and losses of the CNN-LSTM hybrid DL model.

From Figure 11, it can be observed that the training and validation accuracies and losses are dependent on the number of epochs; as the number of epochs increases, it increases the training and validation accuracies and reduces the training and validation losses.

### 4.4. Performance of Proposed CNN-BLSTM Hybrid DL Model

The healthcare industry is experiencing a new transformation thanks to artificial-intelligence technology, especially DL. In the field of disease diagnosis, CNN and BLSTM are two of the most often-used methods. When these algorithms are combined, they form a hybrid model, which is more valuable and versatile than the single CNN and BLSTM models. This Section illustrates the simulation outcomes of the CNN-BLSTM hybrid DL model. Several experiments were conducted utilizing the CNN-BLSTM hybrid deep learning model, with continuous parameter tuning, in order to identify the most suitable parameter values for the model. Table 2 and Figure 6 and Figure 7 illustrate the experimental outcomes of the investigated CNN-BLSTM hybrid DL model in terms of all ML performance measures, while Table 2 and Figure 8 depict the simulation results of the proposed model in terms of all statistical performance measures.

The CNN-BLSTM hybrid deep learning model that was examined demonstrated excellent performance across all machine learning performance metrics. The developed model achieved a classification accuracy of 98.54%, specificity of 97.16%, sensitivity of 100%, precision of 99.00%, recall of 99.00%, AUC score of 97.94%, F1-score of 0.9, and MCC of 0.971, as shown in Table 2 and Figure 6 and Figure 7. The efficiency of the CNN-BLSTM hybrid DL model was also satisfactory in terms of all statistical evaluation metrics. The MSE rate for the investigated model was calculated as 0.0146, the MAE value was observed as 0.0148, and the RMSE value was accounted for as 0.1210, as depicted in Table 2 and Figure 8. The confusion matrix and ROC curve of the CNN-BLSTM hybrid DL model are shown in Figure 9 and Figure 10. The evaluation of a model’s performance primarily relies on two crucial metrics, namely accuracy and loss.

In ML and DL, it is very important to use a model that best fits the nature of the problem. It is not possible to definitively assert the superiority of one model over another without subjecting it to the specific problem at hand. Therefore, the main objective of using multiple models in our study is to find out the most optimum model that best fits the cardiac disease. From Table 2 and Figure 6, Figure 7, Figure 8, Figure 9, Figure 10 and Figure 11, it is quite obvious that the ETCXGB hybrid ML model made 99.98% correct prediction and attained exceptional outcome results for all ML performance metrics. The ETCXGB also performed exceptionally well in terms of statistical performance measures, such as MSE, MAE, and RMSE. Figure 11 depicts the training and validation accuracies and loss of the models.

### 4.5. Performance Comparison of the Utilized Models with Classical ML and DL Schemes

This Section compares the performance of the proposed approach with the earlier methods used for the diagnosis of cardiac disease. For the performance comparison, we took accuracy as the main performance measure for the proposed hybrid models and the classical ML and DL models. Table 3 shows a comparison of the proposed hybrid ML and DL models with the classical ML and DL models.

Table 3 shows that the proposed hybrid models performed better than the earlier ML- and DL-based intelligent computational approaches. Therefore, the proposed scheme is expected to be helpful for both the doctor and patient.

## 5. Conclusions

Chronic diseases, such as Alzheimer’s, cancer, diabetes, cardiac disease, etc., are dangerous and widely spread diseases across the globe, causing millions of deaths every year. Among the chronic diseases, cardiac disease is the most lethal disease, increasing at an accelerating pace across the globe in older people in particular, and has the highest ratio of deaths as compared to other chronic diseases. Smart technologies like ML and DL cannot be ignored in healthcare. These days, hybrid ML and DL approaches work better than classical ML and DL approaches. In this study, we proposed a hybrid ML model named ETCXGB for early diagnosis and prediction of cardiac disease. The UCI Hungarian heart disease dataset was utilized to check the efficiency of the proposed hybrid model. In addition, three hybrid DL models called CNN-RNN, CNN-LSTM, and CNN-BLSTM were also utilized to diagnose cardiac disease. Numerous ML and statistical performance measures were used to analyze the performance of the investigated models. Further, after applying performance measures, the performance of the investigated models was compared, and the highest performance was observed for the ETCXGB hybrid ML model. The performance of the proposed model was also compared with the classical ML and DL methods. By comparing the proposed models with the earlier methods, it was observed that the proposed ETCXGB model enhanced the cardiac diagnosis accuracy by 3.91%; the CNN-RNN improved the prediction accuracy by 1.95%; and the CNN-LSTM and CNN-BLSTM increased the accuracy of the earlier best model by 2.44% and 2.45%, respectively. The simulation outcomes reveal the importance of the proposed ETCXGB hybrid ML model. The limitations of this work include real-time health monitoring of cardiac patients.

## Figures and Tables

**Table 1:** Literature on cardiac disease diagnosis using ML and DL techniques.

Authors	Methodology	Dataset	Accuracy
Amin et al. [25]	Ensemble DT, KNN, SVM, and XGBoost	Ischemia heart disease dataset	94.03%
Ali et al. [26]	LASSO + CNN	NHANES heart disease dataset	79.50%
Abdar et al. [27]	Neural network and Vote (Hybrid model using NB + LR)	UCI Cleveland heard disease dataset	87.40%
Liu et al. [28]	Ensemble DL algorithms	UCI Cleveland heart disease dataset	92.48%
Dwivedi et al. [29]	PS + SVM (SVC, nuSVM, LinSVM)	Z-Alizadeh Sani dataset	93.08%
Jabeen et al. [30]	Stacked KNN	UCI Cleveland heart disease dataset	92.32%
Kumar et al. [31]	LR	UCI Cleveland heart disease dataset	85.00%
Altayeb et al. [32]	MLP	UCI Cleveland heart disease dataset	92.08%
Meyer et al. [33]	NN-GA	Z-Alizadeh Sani dataset	94.10%
Ramalakshmi et al. [34]	Three-tier architecture	UCI Cleveland heart disease dataset	89.96%
Mazhar et al. [35]	Rilef-ETC	UCI Cleveland heart disease dataset	96.09%

**Table 2:** Simulation results of utilized hybrid ML and DL models.

ML Performance Measures								
Model	Accuracy	Sensitivity	Specificity	Precision	Recall	AUC	F1-Score	MCC
ETCXGB	99.98%	100%	99.97%	99.95%	99.96%	99.99%	0.99	0.99
CNN-RNN	98.04%	100%	96.22%	98.00%	98.00%	98.00%	0.98	0.962
CNN-LSTM	98.53%	100%	97.14%	99.00%	99.00%	98.00%	0.99	0.971
CNN-BLSTM	98.54%	100%	97.14%	99.00%	99.00%	98.04%	0.99	0.971
**Statistical Performance Measures**								
**Model**	**MSE**		**MAE**			**RMSE**		
ETCXGB	0.0131		0.0163			0.1143		
CNN-RNN	0.0144		0.0148			0.1200		
CNN-LSTM	0.0121		0.1520			0.1102		
CNN-BLSTM	0.0146		0.0148			0.1210		

**Table 3:** Performance comparison of proposed hybrid models with classical ML and DL models.

Authors	Dataset Used	Scheme	Accuracy
Muhammad et al. [1]	UCI Cleveland heart disease dataset	Rilef-ETC	96.06%
Amin et al. [25]	Ischemia heart disease dataset	Ensemble ML techniques	94.03%
Liu et al. [28]	UCI Cleveland heart disease dataset	Ensembled LSTM	92.48%
Dwivedi et al. [29]	Z-Alizadeh Sani dataset	PS+SVM (SVC, nuSVM, LinSVM)	93.08%
Jabeen et al. [30]	UCI Cleveland heart disease dataset	Stacked KNN	92.32%
Meyer et al. [33]	Z-Alizadeh Sani dataset	Neural network + Genetic Algorithm	94.10%
Barfungpa et al. [37]	UCI Cleveland heart disease dataset	Deep-DenseAquilaNet	99.57%
**This study**	UCI Cleveland heart disease dataset	ETCXGB	99.98%
		CNN-RNN	98.04%
		CNN-LSTM	98.53%
		CNN-BLSTM	98.54%

## Data Availability

The dataset is available online on the Kaggle dataset repository. The implementation code is available on special request to the corresponding author. Also, we have provided it on the Github repository https://github.com/yarkhan007/Heart_Disease-Paper; accessed on 10 December 2024.
